# Dietary Taurine Enhances Intestinal Digestion, Absorption, and Villus Morphology in Mice

**DOI:** 10.3390/ani16101503

**Published:** 2026-05-14

**Authors:** Jingxia Kong, Jianjun Chen, Yu Han, Yuhui Zhang, Shouchuan Jiang, Huahua Du

**Affiliations:** 1 Shulan International Medical College, Zhejiang Shuren University, Hangzhou 310015, China; 2 Zhejiang Key Laboratory of Nutrition and Breeding for High-Quality Animal Products, College of Animal Sciences, Zhejiang University, Hangzhou 310058, China

**Keywords:** taurine, intestinal digestion, nutrient absorption, intestinal stem cell, LPS-induced intestinal injury

## Abstract

Taurine supplementation has been shown to promote growth, but the underlying mechanisms remain incompletely understood. Here, we investigated the effects of taurine on intestinal health in mice under both normal and LPS-induced inflammatory conditions. Thirty-six male C57BL/6J mice were first divided into control and 2% taurine groups for 42 days. On day 43, each group was split into PBS or LPS subgroups, resulting in four groups (Con, LPS, Tau, LPS+Tau). Twelve hours after LPS injection, all mice were euthanized for sample collection. The results demonstrated that taurine supplementation increased body weight gain (*p* < 0.05) without affecting feed intake, indicating improved feed efficiency. Mechanistically, taurine supplementation enhanced intestinal absorptive function through multiple convergent pathways: it increased the activities of digestive enzymes (trypsin, maltase, and sucrase), upregulated nutrient transporter gene expression (*EAAT3*, *SGLT1*, and *FATP4*), and improved villus and microvillus morphology. These functional improvements were confirmed by a 1.7-fold increase in serum D-xylose absorption and significant elevations in total protein and albumin levels (*p* < 0.05). Notably, taurine supplementation upregulated the gene expression of intestinal stem cell (ISC) proliferation markers (*Lgr5* and *Ki67*) and differentiation markers, including *Villin* (enterocyte), *Muc2* (goblet cell) and *ChgA* (enteroendocrine cell). Under LPS challenge, taurine supplementation significantly attenuated disease severity, preserved villus architecture, and reversed inflammation-induced declines in digestive enzymes and nutrient transporters. Collectively, these findings establish taurine as a multifunctional nutrient that enhances intestinal health by optimizing digestive and absorptive capacity while promoting epithelial renewal, thereby supporting its potential application to improve growth and counteract intestinal injury.

## Simple Summary

Taurine, a non-essential sulfur-containing β-amino acid, exhibits diverse biological activities such as blood glucose regulation, bile acid conjugation, blood pressure homeostasis, and antioxidant and anti-inflammatory effects. This study aimed to provide a comprehensive overview of its role in intestinal inflammation. The findings revealed that dietary taurine supplementation markedly promoted nutrient utilization and enhanced intestinal health in mice. These results offer valuable insights for further research into taurine’s protective capacity and therapeutic potential in managing acute inflammatory insults.

## 1. Introduction

Taurine (2-aminoethanesulfonic acid) is a sulfur-containing amino acid that is abundantly present in mammalian tissues and is not incorporated into protein synthesis [1]. Unlike dietary essential amino acids, taurine is conditionally essential, with endogenous synthesis occurring primarily in the liver via cysteine metabolism [2]. However, under certain physiological and pathological conditions, such as rapid growth, inflammatory stress, or intestinal injury, endogenous taurine production may become insufficient to meet metabolic demands, necessitating dietary supplementation [3,4,5]. Accumulating evidence has elucidated that the mechanistic pathways of taurine extend to osmoregulation, membrane stabilization, bile acid conjugation, and anti-inflammatory and antioxidant effects [6,7]. Notably, recent studies from our group and others have shown that taurine supplementation promotes growth performance in various animal models, including mice and piglets, suggesting its potential as a beneficial dietary additive in both agricultural and clinical settings [8,9,10].

The gastrointestinal tract serves as the primary interface between diet and the internal environment, playing a critical role in nutrient digestion and absorption [11]. Optimal intestinal function is essential for efficient nutrient utilization and overall growth. Intestinal absorptive capacity is determined by multiple factors, including the structural integrity of villi and microvilli, the activity of digestive enzymes, and the expression of nutrient transporters on the epithelial surface [12,13]. Disruption of any of these components, as occurs during inflammatory conditions such as lipopolysaccharide (LPS)-induced endotoxemia, leads to malabsorption, impaired growth, and increased disease susceptibility [14]. Therefore, strategies aimed at enhancing intestinal health and accelerating epithelial repair are of considerable interest for both improving growth efficiency and mitigating intestinal injury.

The intestinal epithelium is a rapidly renewing tissue maintained by a pool of intestinal stem cells (ISCs) located at the base of the crypts [15,16]. These Lgr5-positive ISCs continuously proliferate and differentiate into all functional epithelial lineages, including absorptive enterocytes, mucus-secreting goblet cells, and hormone-secreting enteroendocrine cells, thereby sustaining epithelial homeostasis and enabling regeneration following injury [17,18]. Emerging evidence suggests that nutritional interventions can modulate ISC activity to enhance intestinal health [19,20,21]. However, whether taurine exerts its intestinal-protective effects through regulation of ISC dynamics remains largely unexplored.

In the present study, we aimed to investigate the effects of taurine supplementation on intestinal health in mice under both normal physiological conditions and LPS-induced inflammatory stress. We hypothesized that taurine enhances intestinal absorptive function by promoting ISC-mediated epithelial renewal, leading to improved villus architecture, upregulated digestive enzymes and nutrient transporters, and ultimately enhanced growth. These findings provide evidence supporting the potential application of taurine as a dietary strategy to enhance intestinal health.

## 2. Materials and Methods

### 2.1. Experimental Design

A cohort of 36 four-week-old male C57BL/6J mice was obtained from Charles River Laboratories (Beijing, China) and housed three per cage. Following acclimation, the mice were assigned to two initial dietary groups (*n* = 18 per group), matched by comparable body weights: a standard control group (Con) and a group receiving 2% taurine supplementation (Tau). The intervention continued for 42 days, with taurine administered ad libitum via autoclaved drinking water containing 2% taurine [22,23]. On day 43, each initial group was further randomly split into two subgroups (*n* = 9 each). The Con group was divided into a PBS-injected control subgroup (Con) and an LPS-challenged subgroup (LPS). Similarly, the Tau group was subdivided into a taurine-supplemented control subgroup (Tau) and a subgroup receiving both taurine and LPS (LPS+Tau). Mice in the LPS and LPS+Tau subgroups received a single intraperitoneal injection of LPS at 10 mg/kg body weight, while the Con and Tau subgroups were injected with an equal volume of PBS. Twelve hours post-injection, all animals were humanely euthanized for sample collection. The concentration and duration of LPS treatment were adopted from previous studies [24,25], in which this regimen reliably induced a pronounced inflammatory response. We chose a 12-h post-LPS endpoint to evaluate the protective effect of 42-day taurine pretreatment, rather than the resolution phase of inflammation. Prior to euthanasia, blood was collected via retro-orbital puncture. Following cervical dislocation, intestinal tissues were rapidly dissected, snap-frozen in liquid nitrogen, and stored at −80 °C for later analysis. During the entire experiment, all mice were housed under specific pathogen-free (SPF) conditions at Zhejiang University, with unrestricted access to both food and water. The study procedures received ethical approval from the Animal Care Committee of Zhejiang University (No. ZJU20240243).

### 2.2. Body Weight and Diarrhea Index

Body weight and feed intake were monitored by group at 7-day intervals throughout the experiment. Six hours following LPS or PBS administration, fecal consistency was assessed to evaluate diarrhea. The severity of diarrhea was scored based on an established fecal scoring system [26], with the following criteria: 0 for normal, well-formed pellets; 1 for soft but formed feces; 2 for loose, unformed stool; and 3 for watery, projectile discharge. To quantify the overall diarrhea status, a group-level disease activity index (DAI) was calculated for each experimental cohort. The index was derived by summing the individual fecal scores for all mice within a group over the observation period and dividing by the product of the number of mice in the group and the total number of observation days: Diarrhea index = Σ (Group fecal scores)/(Number of mice × Observation days) [27].

### 2.3. Histological Examination of Intestinal Tissue

Samples of the duodenum and jejunum were freshly collected and promptly fixed in 4% paraformaldehyde (3 sections per animal). Following fixation, tissues underwent dehydration through a graded ethanol series, were cleared, and were embedded in paraffin. Sections were sectioned at 5 μm using a microtome. For hematoxylin and eosin (H&E) staining, sections were deparaffinized in xylene and rehydrated in a descending alcohol gradient. They were then stained with hematoxylin for 5 min, briefly differentiated in hydrochloric acid, and blued in ammonia water. Counterstaining was performed with eosin. Finally, the sections were dehydrated, cleared in xylene, and mounted with neutral resin. Stained slides were examined under an optical microscope (Olympus NP-26, Olympus, Beijing, China). Villus height and crypt depth were measured using Image-Pro Plus 6.0 software (Media Cybernetics, Rockville, MD, USA), with a minimum of six intact villus–crypt units evaluated per section [28].

### 2.4. Scanning Electron Microscopy Analysis of Intestinal Microvilli

The morphology of intestinal microvilli was examined using scanning electron microscopy (SEM). The duodenum and jejunum sections (3 sections per animal) were fixed in 2.5% glutaraldehyde for 24 h at 4 °C, followed by post-fixation with 1% osmium tetroxide for 2 h. After fixation, samples were thoroughly washed with phosphate buffer. Dehydration was carried out using a graded ethanol series, with each concentration applied for 15 min, followed by a final treatment with absolute ethanol for 20 min. Subsequently, specimens were immersed in a 1:1 mixture of ethanol and isoamyl acetate for 30 min and then in pure isoamyl acetate for one hour. Critical point drying was performed using a Hitachi HCP-2 system (Hitachi, Tokyo, Japan). Finally, the dried samples were sputter-coated with palladium and imaged under a Philips SU8010 field emission scanning electron microscope (Hitachi, Tokyo, Japan).

### 2.5. Quantitative Real-Time PCR Analysis

Gene expression levels were measured by relative quantitative real-time PCR (qPCR). Briefly, after isolating total RNA with the TransZol Up Kit (#ET111-01-V2; TransGen Biotech, Beijing, China) according to the manufacturer’s instructions, we assessed RNA concentration and purity using a NanoDrop 2000 spectrophotometer (Thermo Fisher Scientific, Waltham, MA, USA). The RNA was then converted to complementary DNA (cDNA) with the Evo M-MLV RT Mix Kit (#AG11728; Accurate Biology, Changsha, China). Real-time PCR was performed on an ABI 7500 system (Applied Biosystems, Foster City, CA, USA) using the quick-start universal SYBR Green Master Mix (#AG11701; Accurate Biology, Changsha, China), and the primer sequences are shown in Table 1. Relative mRNA expression levels of target genes were calculated via the 2^−ΔΔCt^ method, normalized to the endogenous reference gene *β-actin* [29].

### 2.6. Determination of Serum Parameters and Digestive Enzyme Activities

The levels of serum total protein and albumin were determined using an automated biochemistry analyzer (Olympus AU 400, Beckman Coulter, Monsano, Italy). The activities of maltase, sucrase, amylase, trypsin, and lipase in the jejunal contents of mice were measured in strict accordance with the protocols provided in the assay kits. The Maltase Assay Kit (#A082-3-1) and Sucrase Assay Kit (#A082-2-1) were both purchased from Jiancheng Bioengineering Institute (Nanjing, China). The α-Amylase Assay Kit (#G0595W), Trypsin Assay Kit (#G1209W), and Lipase Assay Kit (#G0902W) were all purchased from Grace Biotechnology (Suzhou, China).

### 2.7. Assessment of Intestinal Absorption Capacity

A 10% D-xylose solution was prepared in advance. One hour before sample collection, all mice were administered D-xylose solution by oral gavage at a dosage of 10 mL/kg body weight. Blood samples were subsequently collected, and serum was separated. The D-xylose content in serum was measured strictly according to the instructions of the assay kit from Grace Biotechnology (#G0568W, Suzhou, China).

### 2.8. Statistical Analysis

Each analysis was carried out in duplicate. All data are expressed as the mean ± standard error of the mean. GraphPad Prism (version 8.0.1) was used for all statistical evaluations. A two-tailed unpaired Student’s *t*-test was utilized for comparisons between 2 groups, while two-way ANOVA was employed for comparisons between 4 groups. A *p*-value below 0.05 was considered statistically significant, with significance indicated as * *p* < 0.05 and ** *p* < 0.01.

## 3. Results

### 3.1. Taurine Supplementation Improves Growth and Attenuates Disease Severity

**Figure 1:**
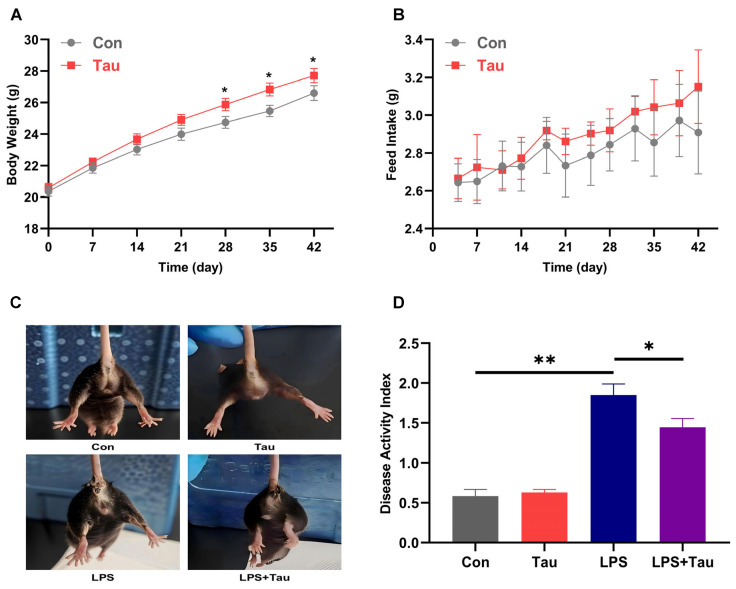
Taurine supplementation improves growth and attenuates disease severity in mice. (**A**) Body weight of mice over a 42-day feeding period (*n* = 18). (**B**) Average daily feed intake of Con and Tau mice (*n* = 18). (**C**) Representative photographs showing diarrhea status of mice. (**D**) Disease activity index (DAI) of mice (*n* = 9). Statistical analysis was performed using a two-tailed unpaired Student’s *t*-test. * *p* < 0.05 and ** *p* < 0.01 show a significant difference.

Since mice were sacrificed 12 h after LPS injection, body weight and feed intake were only evaluated in the control and taurine groups before subgrouping. As the Con and LPS groups (and likewise the Tau and LPS+Tau groups) were subjected to identical experimental conditions prior to sample collection, they were pooled for statistical analysis. Specifically, the Con and LPS groups formed a unified Con group, while the Tau and LPS+Tau groups formed a unified Tau group. The results showed that a significant difference in body weight first emerged on day 28 (*p* < 0.05), with the Tau group exhibiting significantly higher body weight compared to the Con group (Figure 1A). This difference persisted on days 35 and 42. However, no significant difference in feed intake was observed between the Con and Tau groups throughout the entire feeding period (Figure 1B). Diarrheal symptoms, including feces adhering around the anus and matting of perianal fur, were evident in mice 6 h post-LPS challenge from both the LPS and LPS+Tau groups (Figure 1C). In contrast, mice in the Con and Tau groups displayed no apparent abnormalities. Accordingly, no significant difference in DAI was found between the Con and Tau groups (Figure 1D). However, LPS injection resulted in a significant increase in DAI, while the DAI of the LPS+Tau group was significantly lower than that of the LPS group. The results indicated that taurine supplementation conferred benefits in enhancing growth metrics and significantly ameliorating disease severity.

### 3.2. Taurine Supplementation Enhances Intestinal Digestive Function and Nutrient Absorption

**Figure 2:**
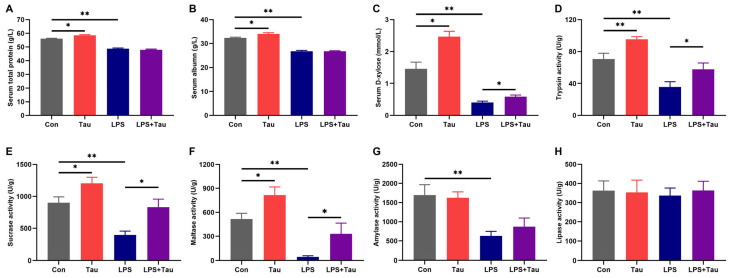
Taurine supplementation enhances intestinal digestive function and nutrient absorption in mice. (**A**,**B**) Serum total protein and albumin levels. (**C**) Serum D-xylose concentration. (**D**–**H**) Activities of trypsin, sucrase, maltase, amylase, and lipase in the jejunum. Statistical analysis was performed using two-way ANOVA. * *p* < 0.05 and ** *p* < 0.01 show a significant difference (*n* = 9).

Compared to the Con group, taurine supplementation in drinking water led to a significant increase in both serum total protein and albumin levels (*p* < 0.05), whereas no significant difference was observed between the LPS and LPS+Tau groups (Figure 2A,B). Elevated serum albumin and total protein levels may be associated with enhanced intestinal digestive enzyme activity and absorptive capacity, although we cannot exclude contributions from other factors such as hepatic protein synthesis, hydration status, or systemic inflammation. Intestinal absorption efficiency was evaluated by measuring serum D-xylose levels after oral administration of a D-xylose solution. Compared with the Con group, serum D-xylose concentration increased by 1.7-fold (*p* < 0.05) in the Tau group but decreased by 72% (*p* < 0.01) in the LPS group (Figure 2C). Notably, serum D-xylose concentration in the LPS+Tau group was 43% higher than that in the LPS group (*p* < 0.05), suggesting that taurine supplementation enhanced intestinal absorption efficiency in mice. Furthermore, compared with the Con group, the activities of trypsin, maltase, and sucrase in the jejunum were significantly increased in the Tau group by 35% (*p* < 0.01), 58% (*p* < 0.05), and 34% (*p* < 0.05), respectively (Figure 2D–F). In contrast, the LPS group exhibited significant (*p* < 0.01) reductions in the activities of trypsin, amylase, maltase, and sucrase. However, taurine supplementation in the LPS+Tau group partially reversed these declines, resulting in trypsin, sucrase, and maltase activities that were 62%, 2.1-fold, and 7.6-fold higher, respectively, than those in the LPS group (*p* < 0.05). No significant difference in amylase and lipase activity was found upon taurine supplementation (Figure 2G,H). Collectively, these findings demonstrated that taurine supplementation enhanced both jejunal digestive enzyme activity and intestinal nutrient absorption capacity in mice.

### 3.3. Taurine Supplementation Up-Regulates the Expression of Intestinal Nutrient Transporters

**Figure 3:**
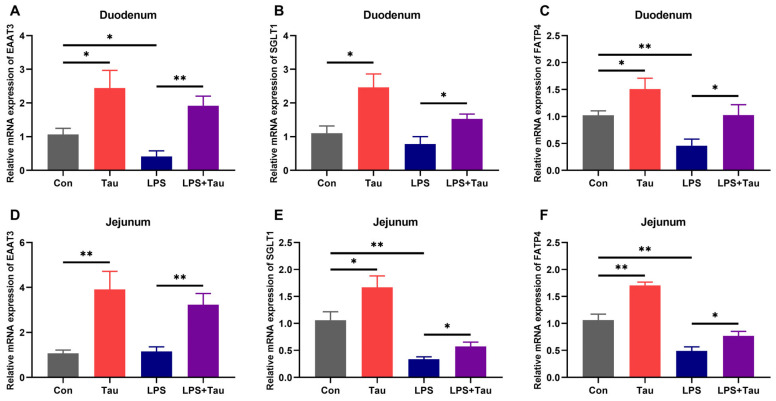
Taurine supplementation upregulates the expression of intestinal nutrient transporters in mice. (**A**–**C**) Relative mRNA levels of *EAAT3*, *SGLT1*, and *FATP4* in the duodenum. (**D**–**F**) Relative mRNA levels of *EAAT3*, *SGLT1*, and *FATP4* in the jejunum. Statistical analysis was performed using two-way ANOVA. * *p* < 0.05 and ** *p* < 0.01 show a significant difference (*n* = 9).

Given that nutrient absorption is mediated by specific transporters [30], we hypothesized that the improvement in absorption following taurine supplementation would correlate with increased expression of these transporters. To test this, we measured the mRNA levels of representative transporters for amino acids (*EAAT3*), glucose (*SGLT1*), and fatty acids (*FATP4*) in the duodenum and jejunum. In the duodenum, compared with the Con group, the expression levels of *EAAT3*, *SGLT1*, and *FATP4* were all significantly (*p* < 0.05) increased in the Tau group by 2.3-fold, 2.2-fold, and 1.5-fold, respectively (Figure 3A–C). In contrast, the LPS group exhibited significant reductions in EAAT3 and FATP4 expression by 61% (*p* < 0.05) and 55% (*p* < 0.01), respectively. However, taurine supplementation in the LPS+Tau group reversed these changes, with expression levels of *EAAT3*, *SGLT1*, and *FATP4* being 4.7-fold (*p* < 0.01), 2.0-fold (*p* < 0.05), and 2.2-fold (*p* < 0.05) higher, respectively, than those in the LPS group. A similar pattern was observed in the jejunum, where taurine supplementation increased the expression of all three nutrient transporters in the corresponding groups (Figure 3D–F). These results demonstrated that taurine supplementation promoted the expression of nutrient transporters in both the duodenum and jejunum, thereby enhancing the transepithelial transport of nutrients.

### 3.4. Taurine Supplementation Ameliorates LPS-Induced Intestinal Injury and Restores Villus Morphology

**Figure 4:**
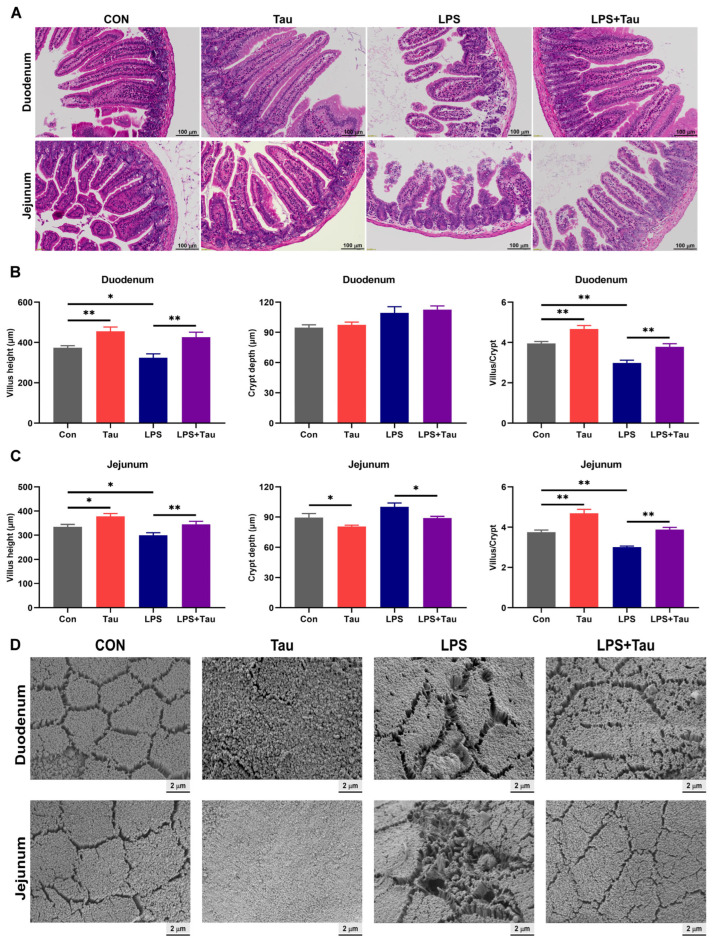
Taurine supplementation ameliorates LPS-induced intestinal injury and restores villus morphology in mice. (**A**) H&E staining of duodenum and jejunum (scale bar = 100 μm). (**B**,**C**) Villus height, crypt depth, and villus height/crypt depth ratio in the duodenum and jejunum (*n* = 9). (**D**) Representative scanning electron micrographs of intestinal microvilli (scale bar = 2 μm). Statistical analysis was performed using two-way ANOVA. * *p* < 0.05 and ** *p* < 0.01 show a significant difference.

Intestinal absorption function is closely associated with villus morphology [31]. Compared with the Con group, the Tau group exhibited superior histological features in the duodenum and jejunum, characterized by more developed and regularly arranged villi with significantly increased villus length (Figure 4A). In contrast, the LPS group showed marked villus injury, including atrophy and shedding in both duodenal and jejunal tissues. Although some damage remained in the LPS+Tau group, clear morphological improvement was observed relative to the LPS group. Quantitative analysis of villus height, crypt depth, and the villus height-to-crypt depth (V/C) ratio further confirmed these observations. In the duodenum, compared with the Con group, the Tau group showed increases of 22% (*p* < 0.01) in villus height and 18% (*p* < 0.01) in V/C ratio, whereas the LPS group exhibited significant decreases in both parameters (Figure 4B). Compared with the LPS group, the LPS+Tau group demonstrated increases of 32% (*p* < 0.01) in villus height and 27% (*p* < 0.01) in V/C ratio. A similar trend was observed in the jejunum, where taurine supplementation significantly increased the villus height and V/C ratio (Figure 4C). SEM observation further revealed that, compared with the Con group, the Tau group had more tightly packed and orderly arranged microvilli in both duodenum and jejunum (Figure 4D). By contrast, microvilli in the LPS group were disorganized and irregular. Importantly, microvilli integrity and alignment were noticeably restored in the LPS+Tau group relative to the LPS group. Collectively, these results demonstrated that dietary taurine supplementation improved duodenal and jejunal morphology, mitigated LPS-induced structural damage, and might thereby contribute to enhanced intestinal absorption.

### 3.5. Taurine Supplementation Promotes Intestinal Stem Cell Activity

**Figure 5:**
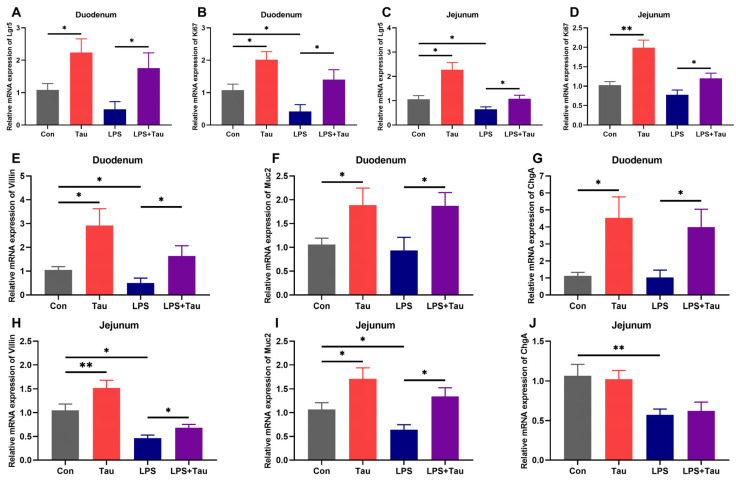
Taurine supplementation promotes the proliferation and differentiation of intestinal stem cells in mice. (**A**–**D**) Relative mRNA levels of intestinal stem cell proliferation markers *Lgr5* and *Ki67* in the duodenum and jejunum. (**E**–**J**) Relative mRNA levels of differentiation markers *Villin*, *Muc2*, and *ChgA* in the duodenum and jejunum. Statistical analysis was performed using two-way ANOVA. * *p* < 0.05 and ** *p* < 0.01 show a significant difference (*n* = 9).

Since taurine upregulates intestinal nutrient transporters and alleviates epithelial damage, we hypothesized that it may act by influencing ISC activity [32]. To test this, we examined proliferation- and differentiation-related gene expression in the intestine. In the duodenum, taurine supplementation significantly (*p* < 0.05) increased the expression of the ISC proliferation marker *Lgr5* by 2.1-fold and *Ki67* by 1.9-fold compared to controls (Figure 5A,B). LPS treatment alone reduced both markers, whereas taurine supplementation in LPS-treated mice (LPS+Tau) reversed these changes, elevating *Lgr5* and *Ki67* expression by 3.6-fold and 3.4-fold (*p* < 0.05), respectively, relative to LPS alone. A similar pattern was observed in the jejunum (Figure 5C,D). We next assessed the gene expression of differentiation markers of ISC. In the duodenum, taurine supplementation significantly (*p* < 0.05) upregulated the enterocyte marker *Villin*, the goblet cell marker *Muc2*, and the enteroendocrine cell marker *ChgA* by 2.8-fold, 1.8-fold, and 4.1-fold, respectively, versus controls (Figure 5E–G). LPS treatment reduced the mRNA level of *Villin* by 53% (*p* < 0.05), while taurine supplementation in the LPS+Tau group increased *Villin*, *Muc2*, and *ChgA* expression by 3.3-fold, 2.0-fold, and 3.9-fold, respectively, compared to LPS alone (*p* < 0.05). Similar effects on *Villin* and *Muc2* expression were observed in the jejunum (Figure 5H,J). Together, these findings indicated that taurine promoted both the proliferation and differentiation of ISCs.

## 4. Discussion

The present study demonstrates that taurine supplementation exerts beneficial effects on intestinal health in mice, particularly under inflammatory conditions induced by LPS challenge. Our findings reveal that taurine enhances feed efficiency, attenuates disease severity, and improves intestinal absorptive function through multiple complementary mechanisms, including upregulation of digestive enzyme activity, enhancement of nutrient transporter expression, preservation of villus morphology, and promotion of ISC proliferation and differentiation.

A key finding of this study is that taurine supplementation alone, without any inflammatory challenge, significantly improved body weight gain and intestinal morphology in healthy mice. Consistent with our observations, previous studies have reported that dietary taurine enhances growth performance in piglets [8] and broilers [33], and improves intestinal mucosal architecture across species [34,35]. The Tau group exhibited increased villus height and V/C ratio, accompanied by more tightly packed microvilli-structural features closely associated with enhanced nutrient absorption [36]. These improvements were paralleled by enhanced digestive enzyme activities (trypsin, maltase, sucrase) and upregulated expression of nutrient transporters (*EAAT3*, *SGLT1*, *FATP4*), which are direct determinants of amino acid, glucose, and fatty acid absorption [37]. The functional significance was confirmed by elevated serum total protein and albumin levels, markers of systemic protein nutritional status, as well as increased D-xylose absorption, a validated indicator of intestinal absorptive capacity. Collectively, these observations suggest that taurine acts as a trophic factor that optimizes intestinal architecture and function even under basal conditions, which may explain the sustained growth advantage observed in taurine-supplemented mice from day 28 onward.

More importantly, taurine supplementation significantly mitigated LPS-induced intestinal injury and dysfunction. LPS challenge resulted in severe villus atrophy and shedding, disorganized microvilli, reduced digestive enzyme activities, and downregulated nutrient transporter expression—all of which contribute to malabsorption and increased disease severity. Taurine pretreatment in LPS-challenged mice partially reversed these pathological changes, restoring villus height, microvilli integrity, enzyme activities, and transporter expression. The functional recovery was evidenced by improved D-xylose absorption and significantly lower disease activity index scores in the LPS+Tau group compared to LPS alone. It is important to clarify that our experimental design assessed the protective capacity of taurine against acute inflammatory injury, not its therapeutic effect on established inflammation. The 12-h time point, which corresponds to peak inflammation based on prior reports, allowed us to determine whether chronic taurine supplementation attenuates the inflammatory response at its early stage, as evidenced by preserved villus architecture and maintained digestive function in the LPS+Taurine group compared to the LPS group. These findings position taurine as a promising nutritional intervention for protecting intestinal structural integrity during inflammatory insults. Taurine’s restoration of villus architecture under LPS challenge may be mediated through regulation of gut microbiota, such as promoting butyrate-producing bacteria that reinforce tight junctions and reduce inflammation [38].

The mechanisms underlying taurine’s protective effects appear to involve regulation of ISC dynamics. Our data show that taurine supplementation significantly increased expression of *Lgr5* and *Ki67*, markers of ISC proliferation, while also upregulating differentiation markers for all major intestinal epithelial lineages—enterocytes (*Villin*), goblet cells (*Muc2*), and enteroendocrine cells (*ChgA*). This broad effect on ISC activity suggests that taurine promotes both the expansion of the stem cell pool and its subsequent differentiation into functional epithelial cells [39]. The enhanced ISC activity likely contributes to the improved villus morphology and accelerated epithelial repair observed in taurine-treated mice, particularly following LPS-induced damage. This stem cell-mediated mechanism represents a novel perspective on how taurine supports intestinal homeostasis and regeneration.

Notably, the consistency between our mouse findings and previous piglet trials strengthens the translational relevance of these observations [8]. The convergence of results across species suggests that taurine’s effects on intestinal health may represent a conserved biological mechanism. Furthermore, the absence of significant differences in feed intake between Con and Tau groups throughout the study indicates that the growth-promoting effects of taurine are attributable to enhanced nutrient absorption efficiency rather than increased consumption—a particularly valuable characteristic for agricultural applications where feed conversion efficiency is paramount.

Several limitations of this study should be acknowledged. First, while we observed significant changes in nutrient transporter and intestinal stem cell gene expression, protein-level validation was not performed, leaving the translational regulation of these genes unclear. Second, the gut microbiota was not analyzed. Given that taurine is known to be metabolized by certain bacteria into bioactive compounds such as hydrogen sulfide, the potential contribution of microbial modulation to the observed effects cannot be excluded. Third, the study duration (42 days of taurine supplementation plus acute LPS challenge) was relatively short, and the long-term effects of taurine on intestinal health remain unknown. Future studies incorporating proteomic analysis, microbiota profiling, and extended supplementation periods are needed to further validate these findings.

## 5. Conclusions

Our study provides integrated evidence that taurine supplementation coordinately enhances digestive, absorptive, and epithelial renewal functions. We demonstrate that taurine not only improves baseline intestinal physiology—as evidenced by increased enzyme activities, nutrient transporter expression, and villus morphology—but also confers resilience against acute inflammatory injury. Critically, the novel finding that taurine upregulates ISC markers suggests an active mechanism involving epithelial turnover, rather than passive protection alone. Together, these findings provide a mechanistic foundation for exploring taurine in other models of intestinal inflammation.

## Figures and Tables

**Table 1:** Forward and reverse primer sequences for PCR analysis.

Gene	Primer Sequences (5′ to 3′)		GenBank Accession
*β-actin*	F: CCACCATGTACCCAGGCATT	R: AGGGTGTAAAACGCAGCTCA	NM_007393.5
*EAAT3*	F: CTTCCTACGGAATCACTGGCT	R: CGATCAGCGGCAAAATGACC	NM_009199.3
*SGLT1*	F: CTGCCCATGTTCCTCATGGT	R: TGGTGTGCCGCAGTATTTCT	NM_019810.4
*FATP4*	F: ACTGTTCTCCAAGCTAGTGCT	R: GATGAAGACCCGGATGAAACG	NM_011989.5
*Lgr5*	F: CAGCCTCAAAGTGCTTATGCT	R: GTGGCACGTAACTGATGTGG	NM_010195.2
*Ki67*	F: ATCATTGACCGCTCCTTTAGGT	R: GCTCGCCTTGATGGTTCCT	XM_006507413.5
*Villin*	F: TCAAAGGCTCTCTCAACATCAC	R: AGCAGTCACCATCGAAGAAGC	NM_009509.2
*ChgA*	F: ATCCTCTCTATCCTGCGACAC	R: GGGCTCTGGTTCTCAAACACT	NM_007693.2
*M* *uc* *2*	F: AGGGCTCGGAACTCCAGAAA	R: CCAGGGAATCGGTAGACATCG	NM_023566.4

*EAAT3* = excitatory amino acid transporters 3; *SGLT1* = Sodium-glucose cotransporter 1; *FATP4* = fatty acid transport protein 4; *Lgr5* = Leucine-rich G repeat-containing protein-coupled receptor 5; *Ki67* = Kiel 67; *ChgA* = Chromogranin A; *Muc2* = mucin 2.

## Data Availability

The data used to support the findings of this study are available from the corresponding author upon request.

## Abbreviations

The following abbreviations are used in this manuscript:

BW	Body weight
DAI	Disease activity index
H&E	Hematoxylin and eosin
ISC	Intestinal stem cell
LPS	Lipopolysaccharide
SEM	Scanning electron microscopy
